# Some Considerations about Pornography Watching in Early Adolescence

**DOI:** 10.3390/ijerph191710818

**Published:** 2022-08-30

**Authors:** Luca Cerniglia, Silvia Cimino

**Affiliations:** 1 Faculty of Psychology, International Telematic University Uninettuno, 00186 Rome, Italy; 2 Department of Dynamic, Clinical and Health, Sapienza University of Rome, 00185 Rome, Italy

## 1. Introduction

Adolescence is a time of significant transition because of the rapid acceleration of bodily changes [1]. This developmental stage, however, is not merely characterized by physical modifications; it is also characterized by cognitive, social/emotional, and interpersonal changes in young people [2]. While these processes are supported by the maturation of neurobiological areas of the brain, such as the prefrontal cortex (which is involved in decision-making, impulse control, and long-range planning), youths are impacted by external elements, such as their environment, culture, peers, and the media [3]. During their development, adolescents move toward independence and future interests, they experience increased risk taking and cognitive growth, and become interested in sexuality. The new interest towards sexuality (even in early adolescence: 11–13 years of age) brings more consideration for their own privacy, produces experiments with their bodies (masturbation), and urges comparisons with peers about their body shape and characteristics [4]. If, on one hand, after puberty, adolescents experience a drive towards sexuality, on the other hand, this very drive and the changes in youths’ bodies can constitute a motive of anxiety and preoccupation and may even be perceived as traumatic, due to its intensity [5]. According to several authors, the new sexual body is not fully integrated in youths until the emerging adulthood developmental phase and masturbation and the first approaches with peers are of crucial importance in the realization of this complex process of integration [6]. Moreover, it has been authoritatively stated that during adolescence, the psychic equilibrium is in a state of fluidity and changeability where nothing is yet firmly established, so that first passions and juvenile infatuations are defined more as narcissistic identifications rather than object relationships [7]. In this complex landscape, witnessing crucial developmental touchpoints, adolescents historically experienced a gradual approach to sexuality, generally proceeding in subsequent steps from platonic love to first kiss, petting, manual and/or oral sex, and intercourse. The construction of their own body image, the perception of others as desirable sexual partners, and the experience of what sensations sexual interactions arise, were long-lasting processes, starting with puberty (around 11–12 years), continuing until emerging adulthood, mainly relying on peer modelling and comparison of experiences [8].

### 1.1. What Is Happening Today?

Research in this field predominantly portrays pornography consumption as a way adolescents nowadays choose to explore sexuality and as something that is becoming normative [9].

In a way, this literature is parallel to that focusing on Internet use and misuse. Some years ago, most publications concentrated on the possible risks linked to the use of the Web. Then, as being online has become ubiquitous, two different branches of literature emerged. One focused on the many positive effects of being online (e.g., finding useful information easily, keeping in touch with friends and relatives, learning online, etc.) and one still concentrated on its possible negative effects. In the latter case, however, most (almost the totality) of the literature considered the possible risks of *excessive* use of the internet so that *normative* use is very rarely posited as a problematic activity, even in very young individuals [10,11]. In previous studies, orientated by the Developmental Psychopathology framework, we tried to raise the argument that Internet use in young subjects poses a number of risks, regardless of how often and how long the Web is used [12]. In the case of pornography consumption, the same pattern appears to delineate (in our opinion), with several studies reporting pornography use by young adolescents as a cultural/social phenomenon, almost ignoring possible risks, if not in the case of *excessive* use [13].

### 1.2. Two Crucial Questions

We believe that this approach could be accepted, provided that one could answer positively to at least two questions: (1) Can pornography be considered a sort of informative material useful to early adolescents to understand sexuality? (2) Is the content youths can watch controlled by anyone to prevent them viewing violent sex and other forms of sexuality that could be particularly perturbating? We think the answer to these questions is “no” or, more prudently, “partially”. Pornographic movies online are not constructed as informative materials, rather as a form of adult entertainment. Most of this material is intended to arouse the watcher as much as possible, in a short period of time, following unrealistic scripts, by showing unrealistic bodies and sexual behaviors [14]. It would be like watching a movie from the “Fast and Furious” series with the aim of learning to drive.

While an adult is generally capable of distinguishing fiction from reality and should be able to watch porn to *fantasize* about being part of the scene, an early adolescent has barely begun to develop an abstract thought and cannot make this difference effectively [15]. Thus, the risk is that very young youths are confronted with a supernormal stimulus they are not structurally and functionally ready to handle [16]. On the other hand, imagination, which should be cultivated in adolescence [17], could be hindered by hyper-explicit pornographic material (that is explicit by definition).

### 1.3. Conclusions

It is our opinion that the possibility of watching pornographic material by early adolescents should be carefully considered rather than passively accepted as something inevitable due to the ubiquitous use of the internet.

## Data Availability

Not applicable.
